# Hydrogen Isotopic Fractionation in the Solar Nebula and Implications for Water in Earth and Inner Solar System Bodies

**DOI:** 10.1021/acsearthspacechem.6c00183

**Published:** 2026-09-24

**Authors:** Abu Asaduzzaman

**Affiliations:** School of Science, Engineering and Technology, 43324 Penn State Harrisburg, Middletown, Pennsylvania 17057, United States

**Keywords:** Water, Deuterium, Adsorption, Earth, Mars, Carbonaceous chondrites, Vesta, Density functional theory

## Abstract

The origin of Earth’s water requires explanation of both its abundance and its elevated D/H ratio relative to the solar nebula. Previous work (Asaduzzaman et al., 2015) demonstrated that dissociative chemisorption of water on olivine grains in the solar nebula could supply several ocean equivalents of water to the growing Earth. However, this mechanism has been challenged on isotopic grounds, as it does not inherently explain the enrichment of D/H ratios relative to solar values. Here, I quantify hydrogen isotopic fractionation between nebular gas and a set of condensed phases using a combination of quantum chemical calculations and statistical thermodynamics. Equilibrium fractionation factors are determined as functions of temperature for (i) chemisorbed hydroxyls on olivine surfaces, (ii) structural hydroxyls in apatite and pyroxenes (including pigeonite), and (iii) representative organic compounds. The results show that deuterium is preferentially partitioned into condensed phases, with enrichment factors increasing systematically with decreasing temperature. The calculated enrichment factors reach values comparable to those observed in terrestrial water, carbonaceous chondrites, Mars, and asteroid Vesta at temperatures of ∼175–300 K. Kinetic constraints based on hydrogen diffusion in olivine indicate that isotopic equilibration between gas and grain surfaces is plausible within the lifetime of the solar nebula. These findings demonstrate that equilibrium gas–grain isotopic fractionation can naturally generate elevated D/H ratios in water adsorbed onto silicate grains. This study offers an explanation to prior criticism of adsorption-based models by showing that nebular water can acquire terrestrial-like isotopic compositions through low-temperature equilibration with grain surfaces. Chemisorption-mediated accretion therefore provides a plausible complementary mechanism for explaining both the abundance and isotopic composition of water in Earth and other inner solar system bodies, and may reduce part of the required contribution from carbonaceous chondrites.

## Graphical abstract

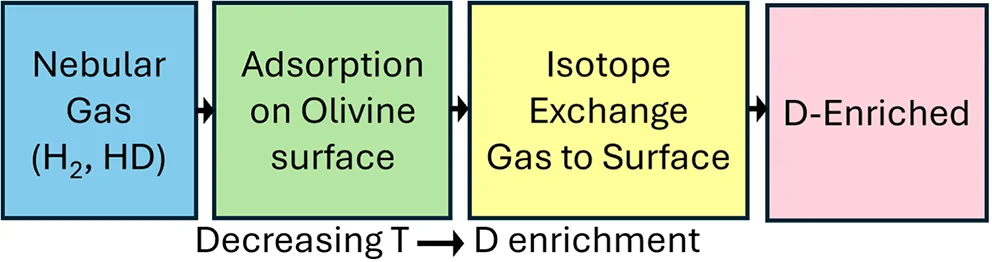

## 1 Introduction

The origin of water on Earth remains one of the most fundamental problems in planetary science, with broad implications for the formation of habitable environments in the solar system. Any viable model must account not only for the abundance of water but also for its isotopic composition, particularly the elevated D/H and ^15^N/^14^N ratios relative to the solar nebula. The D/H ratio of Earth’s mantle is enriched by a factor of ∼4–6 compared to solar values, while ocean water exhibits even greater (∼7–8) enrichment. Part of the latter likely reflects atmospheric hydrogen loss followed by D–H exchange between hydrogen gas and water vapor during ocean formation.^1^

Recent isotopic studies have provided new constraints on the origin of terrestrial water. Analyses based on noble gas isotopes suggest that a significant fraction of Earth’s water may have been incorporated during accretion, with evidence for heterogeneous water distribution between the upper and lower mantle. In addition, hydrogen isotopic compositions of samples from Baffin Island and Icelandic lavas, which are relatively unaffected by surface water recycling, indicate the presence of a solar hydrogen component in the deep mantle.^2^ More recent work on enstatite chondrite–like materials suggests that the building blocks of Earth may have been intrinsically rich in hydrogen, challenging the traditional view that water was primarily delivered by external sources such as carbonaceous chondrites.^3–7^

Several hypotheses have been proposed to explain the origin of Earth’s water. The most widely discussed models invoke delivery by carbonaceous chondrite like materials, based on the similarity of their D/H ratios to that of terrestrial water. However, this scenario raises unresolved questions regarding the origin of water in the parent bodies themselves and the mechanisms that established their isotopic signatures. Enstatite chondrites have also been proposed as plausible building materials for Earth based on their isotopic affinities, although their highly reducing formation conditions imply that hydrogen must have been subsequently oxidized to form water before ∼3.8 Ga. If carbonaceous chondrites supplied most of Earth’s water, one must still explain how these meteorites acquired water with Earth-like isotopic compositions. A growing body of evidence suggests that both Earth and meteorite parent bodies may have inherited their water through common nebular processes rather than through late-stage delivery alone.^5^

An alternative mechanism, originally proposed by Drake and co-workers and subsequently developed quantitatively by Asaduzzaman et al.,^8^ involves direct incorporation of water into silicate grains through adsorption from the solar nebula. That study demonstrated that dissociative chemisorption of water on olivine surfaces, followed by diffusion of hydrogen into the grain interior, could supply several ocean equivalents of water during planetary accretion. This model provides a physically plausible pathway for the in situ incorporation of nebular water into growing planetary bodies, without requiring large-scale transport from distant regions of the protoplanetary disk.

Despite its success in explaining the abundance of terrestrial water, the adsorption-mediated accretion model has been questioned on isotopic grounds. Specifically, it has been argued that water derived directly from the solar nebula should retain a near-solar D/H ratio, significantly lower than that observed for Earth and other inner solar system bodies. Observations of solar wind and primitive materials, as well as isotopic measurements across planetary bodies, have been interpreted as inconsistent with a purely nebular origin of terrestrial water. However, recent experimental and theoretical studies demonstrate that multiple processes in protoplanetary disks including photodissociation of water isotopologues and gas–grain chemical interactions, can generate substantial hydrogen isotopic fractionation, leading to spatial and temporal heterogeneity in D/H ratios.^9,10^

This criticism neglects a key aspect of hydrogen isotope geochemistry: equilibrium isotopic fractionation between gas and condensed phases. Deuterium preferentially partitions into condensed phases relative to hydrogen, and the magnitude of this fractionation increases strongly with decreasing temperature. Such gas–grain isotopic exchange is well documented in interstellar and protostellar environments, where grain-surface chemistry plays a central role in controlling isotopic compositions. In addition, studies of meteoritic organic matter show substantial deuterium enrichment consistent with formation and processing in low-temperature environments involving gas–grain interactions. These observations suggest that isotopic exchange between nebular gas and solid phases could have significantly modified the D/H ratio of water incorporated into planetary building materials.^11–13^

Several additional mechanisms have been proposed to explain hydrogen isotopic enrichment in planetary materials, including ion–molecule chemistry in molecular clouds, photochemical self-shielding, radial transport within protoplanetary disks, inheritance from interstellar reservoirs, and low-temperature ice chemistry. The mechanism explored here should not be viewed as mutually exclusive with these processes. Instead, equilibrium gas-grain fractionation may have operated alongside them and contributed to the final D/H inventory of planetary materials.

In this study, I investigate the extent to which equilibrium gas–grain isotopic fractionation could have modified the D/H ratio of water incorporated into silicate grains during nebular accretion. Using a combination of quantum chemical calculations and statistical thermodynamics, I evaluate hydrogen isotopic fractionation between nebular gas species and (i) chemisorbed hydroxyl groups on olivine surfaces, (ii) structural hydroxyls in apatite, pyroxenes, and pigeonite, and (iii) representative organic compounds. Recent first-principles studies of isotope partitioning in silicate minerals further highlight the importance of local bonding environments and zero-point energy effects in controlling isotopic preference, reinforcing the relevance of atomistic modeling approaches. These calculations enable direct assessment of whether adsorption-mediated water incorporation can reproduce the observed isotopic compositions of Earth and other inner solar system bodies.^14^

In addition to thermodynamic constraints, I also examine the kinetic feasibility of isotopic equilibration under nebular conditions, based on available data for hydrogen diffusion in silicate minerals. Experimental and modeling studies indicate that hydrogen diffusion and exchange in olivine can lead to significant isotopic modification on geologically relevant time scales, even at relatively low temperatures. This allows evaluation of whether equilibrium fractionation could have been attained within the lifetime of the solar nebula.^15^

## 2 Thermodynamic and Quantum Chemical Methods

### 2.1 Background and Framework

The present study builds on our earlier work on chemisorption-mediated incorporation of water into olivine in the solar nebula (Asaduzzaman et al.)^8^ In that study, dissociative adsorption of water on olivine surfaces was quantified using a Langmuir adsorption framework to estimate the total amount of water that could be incorporated into accreting planetary bodies. In addition, equilibrium abundances of deuterium-bearing gas species were evaluated at nebular conditions (500–900 K, ∼10^–3^ bar) using free-energy minimization with the Thermo-Calc software^16^ and thermochemical data from the JANAF tables.^17^

Those calculations showed that hydrogen deuteride (HD) is the dominant deuterium-bearing species under nebular conditions and that its relative abundance is only weakly dependent on temperature over this range. Based on these results, the present work focuses on equilibrium isotopic exchange between nebular hydrogen gas and condensed phases containing hydroxyl groups.

Consistent with our previous studies on hydrogen isotope fractionation in mineral–fluid systems,^18,19^ isotopic fractionation is treated within a statistical thermodynamic framework based on molecular partition functions.

### 2.2 Isotopic Exchange Reaction and Fractionation Factor

Hydrogen isotopic fractionation between nebular gas and chemisorbed hydroxyl groups on olivine surfaces is described by the exchange reaction

a $${\left(\mathrm{OH}\right)}_{\mathrm{surf}}+{\left(\mathrm{DH}\right)}_{\mathrm{gas}}↔{\left(\mathrm{OD}\right)}_{\mathrm{surf}}+{(H_{2})}_{\mathrm{gas}}$$

At equilibrium

1 $$K_{a}\left(T\right)=\frac{{\left(OD/OH\right)}_{surf}}{{\left(DH/H_{2}\right)}_{gas}}=\frac{{\left(D/H\right)}_{surf}}{{2\left(D/H\right)}_{gas}}$$

where *K*_a_ is the equilibrium constant for the reaction (a) and the terms within the parentheses indicate ratios of mole numbers of the indicated species.

The D/H enrichment factor between condensed and gas phases is defined as

2 $$\alpha =\frac{{\left[\frac{D}{H}\right]}_{condensed}}{{\left[\frac{D}{H}\right]}_{gas}}$$

Following standard isotope thermodynamics, the enrichment factor is related to the equilibrium constant by

3 $$a=2K_{a}$$

### 2.3 Statistical Thermodynamic Formulation

The equilibrium constant is calculated using molecular partition functions

4 $$K_{a}\left(T\right)=\prod_{i}{\left(\frac{q_{i}^{*}}{q_{i}}\right)}^{v_{i}}$$

where *q*_*i*_ and *q*_i_^*^ are the partition functions of the light and heavy isotopologues, respectively, ν_*i*_ is the stoichiometric coefficient of species *i* in the exchange reaction and π is the conventional product symbol.

At the temperatures relevant to the solar nebula, the partition function for each species can be approximated as a product of translational, rotational, and vibrational contributions. For gas-phase molecules, all three contributions are included, whereas for condensed-phase species (chemisorbed or structural hydroxyls), only vibrational contributions are relevant.

Under the harmonic oscillator and rigid rotor approximations, rotational and translational terms largely cancel in isotope ratios, and the equilibrium constant reduces to a function of vibrational frequencies. This formulation yields the reduced partition function ratio (RPFR), commonly used in isotope geochemistry

5 $$K_{a}=\frac{({q^{*}/q)}_{surf}}{({q^{*}/q)}_{gas}}=\frac{\Pi {\left[\frac{\nu^{*}}{\nu }\left(\frac{e^{-u_{j}^{*}/2}}{e^{{-u}_{j}/2}}\right)\left(\frac{{1-e}^{-u_{j}}}{{1-e}^{{-u}_{j}^{*}}}\right)\right]}_{condensed}}{\frac{1}{2}\Pi {\left[\frac{\nu^{*}}{\nu }\left(\frac{e^{-u_{j}^{*}/2}}{e^{{-u}_{j}/2}}\right)\left(\frac{{1-e}^{-u_{j}}}{{1-e}^{{-u}_{j}^{*}}}\right)\right]}_{gas}}$$

where the asterisk indicates a heavier isotopic species, the factor 1/2 in the denominator after the second equality stands for the ratio of the symmetry numbers of HD (σ = 1) and H_2_ (σ = 2) and *u*_*j*_ *= h*v_*j*_/*kT (k* is Boltzmann constant and v_*j*_ are the vibrational frequency of species).

This approach follows directly from the formalism developed by Urey^20^ and has been successfully applied in our previous studies of hydrogen isotope fractionation in silicate systems.^18,19^

### 2.4 Quantum Chemical Calculations

A workflow for the calculation of isotope fractionation has been illustrated in Figure 1 and discussed in detail in the following.

**Figure 1:**
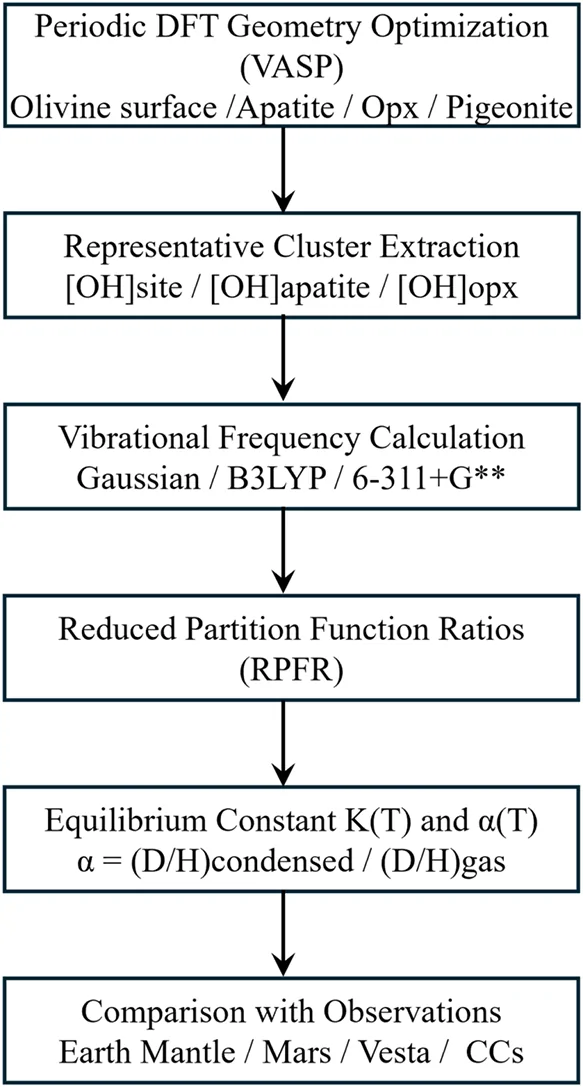
Workflow for the calculations. The same procedure has been used in our previous studies.^18,19^

#### 2.4.1 Structural Models

The atomic structures of hydroxylated olivine surfaces and bulk mineral phases (apatite, orthopyroxene, and low-Ca clinopyroxene, i.e., pigeonite) were first optimized using density functional theory (DFT) with the VASP code.^21,22^ Calculations employed:

- PBE exchange–correlation functional.^23^
- Projector augmented-wave (PAW) method.^24,25^

- Plane-wave cutoff energy of 450 eV.
- Monkhorst–Pack *k*-point grids:^26^
  - 5 × 5 × 5 for bulk phases.
  - 2 × 2 × 1 for surface slabs.

These modeling strategies are consistent with those used in our earlier adsorption studies.^8,27^

The olivine surface model was based on the hydroxylated forsterite (010) surface previously investigated by Asaduzzaman et al.^8,27^ The slab consisted of 8 atomic layers separated by a vacuum region of 15 Å. Water dissociatively adsorbs onto exposed Mg–O surface sites, producing surface hydroxyl groups corresponding to approximately a monolayer coverage.

#### 2.4.2 Cluster Extraction and Vibrational Analysis

From the optimized periodic structures, finite stoichiometric cluster models representing local bonding environments around hydroxyl groups were constructed (Figure S1). In these clusters, the OH (or OD) group was positioned centrally to minimize boundary effects. All clusters are charged neutral with singlet spin state. Terminal atoms at the cluster boundaries were fixed to preserve the periodic geometry.

Vibrational frequency calculations were then performed using the Gaussian^28^ software package at the:

- B3LYP level of theory.^29,30^

- 6–311+G** basis set.

Vibrational calculations were performed after relaxation of atoms directly associated with the OH/OD group and its first-neighbor coordination shell. and both optimization and frequency calculations were carried out for molecular species, including gas-phase H_2_, HD, and representative organic compounds (benzene and ethylbenzene). No imaginary frequencies were obtained, confirming that all structures correspond to true minimum on the potential energy surface.

Finite cluster models were used because they provide a computationally tractable representation of the local bonding environment around the isotopically substituted H/OH sites while retaining the vibrational properties that control equilibrium isotope fractionation. This hybrid periodic–cluster approach follows the methodology established in our prior isotope fractionation studies,^18,19^ where it has been shown to reproduce experimentally determined fractionation factors with high accuracy. Further, previous studies show hydrogen isotope fractionation factors are relatively insensitive to modest changes in functional and basis set. Nevertheless, systematic uncertainties arising from the B3LYP/6–311+G** approach are expected to be on the order of a few percent.

The mode of vibrations of all condensed phases and the gaseous molecules along with corresponding available experimental values are shown in Table S1.

### 2.5 Application to Different Phases

The above methodology was applied to three classes of condensed phases:

1. Chemisorbed hydroxyls on olivine surfaces (Figure 2)
  representing water incorporated during nebular adsorption.
2. Structural hydroxyls in minerals
  including apatite, orthopyroxene, and pigeonite, with the exchange reaction reformulated by replacing lattice-bound OH group by OD.
3. Organic compounds, hydrogen isotope substitution was modeled in:
  - aromatic C–H sites (benzene).
  - benzylic C–H sites (ethylbenzene).

**Figure 2:**
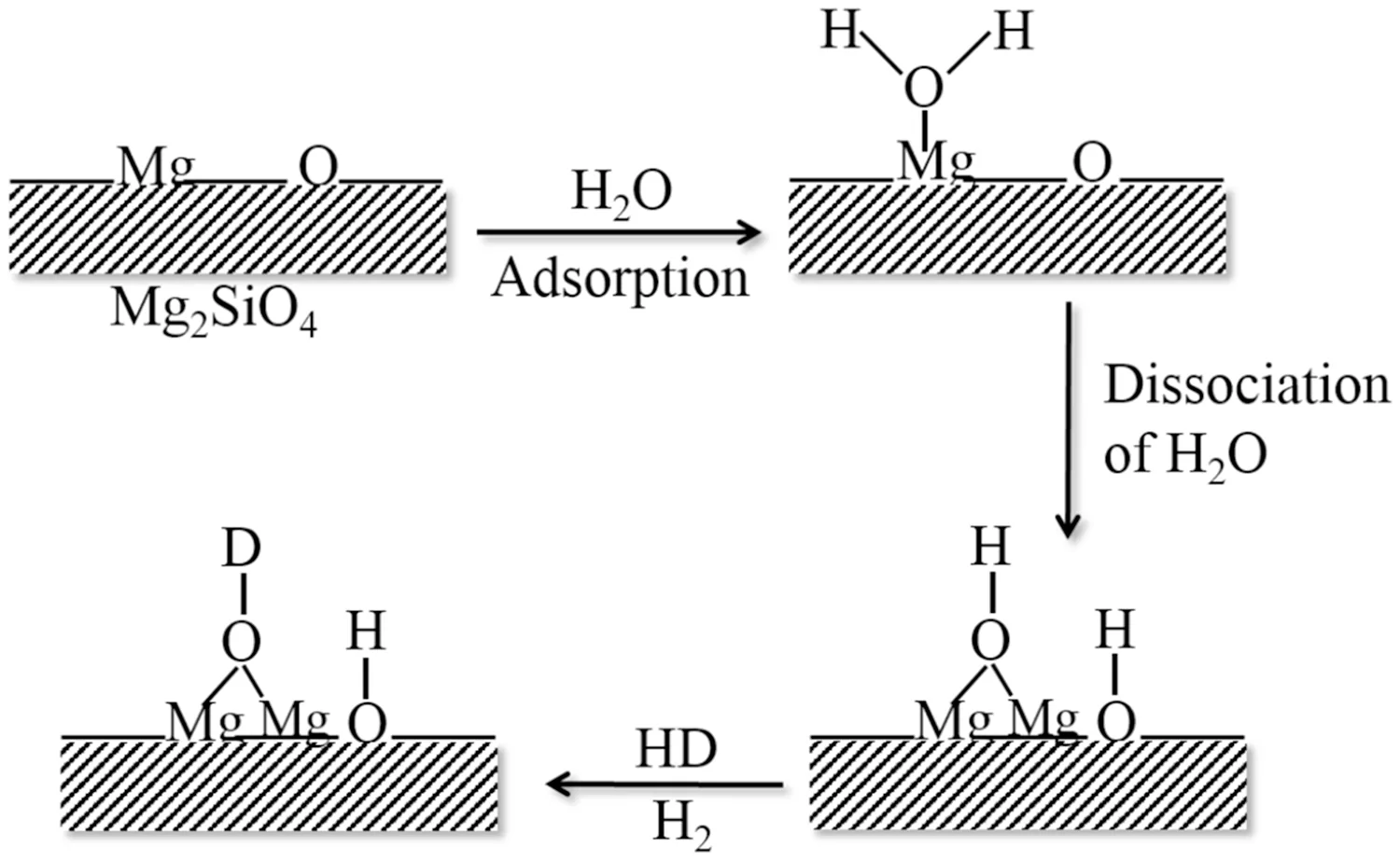
Schematic representation of dissociative adsorption of water on olivine surface followed by D/H exchange.

These systems were selected to represent major hydrogen reservoirs in planetary materials and meteoritic organics.

Benzene and ethylbenzene were selected as simplified end-member models representing aromatic and benzylic hydrogen sites. While meteoritic IOM contains substantially greater structural complexity, including aliphatic, heteroatom-bearing, and cross-linked components, these two model compounds capture the principal bonding environments expected to dominate hydrogen isotope partitioning.

### 2.6 Calculation of Fractionation Factors

The vibrational frequencies obtained from quantum chemical calculations were used to evaluate RPFRs and equilibrium constants (eqs 4–6). These were then used to calculate isotopic enrichment factors (α) as functions of temperature for condensed phases. For organic molecules, the free energy of reaction was calculated directly from Gaussian. The calculated fractionation factors are subject to uncertainties associated with the choice of DFT functional, basis set, and cluster representation. Based on previous DFT studies of equilibrium isotope fractionation,^18,19^ these systematic uncertainties are expected to be on the order of a few percent and will not affect the overall temperature dependence or the main conclusions of this work.

The resulting α–*T* relationships provide a direct quantitative basis for comparing hydrogen isotope fractionation in different condensed phases with observed D/H ratios in planetary materials.

## 3 Results and Discussion

### 3.1 Thermodynamic Behavior of Hydrogen Isotopic Fractionation

The calculated equilibrium enrichment factor, α, exhibits a systematic dependence on temperature for all condensed phases considered in this study. In every case, deuterium is preferentially partitioned into the condensed phase, and the enrichment factor increases with decreasing temperature. This behavior reflects the lower zero-point energy of O–D relative to O–H bonds, which stabilizes deuterium-bearing species in solids at low temperature, consistent with fundamental principles of isotope thermodynamics and prior theoretical analyses of hydrogen isotope fractionation.^12,14^

The calculated α–*T* relations for chemisorbed hydroxyls on olivine surfaces, hydrocarbons, and structural hydroxyls in silicate minerals are shown in Figures 3, 4, and 5. Despite differences in bonding environments, these phases exhibit broadly similar temperature dependences, indicating that hydrogen isotope fractionation in the solar nebula is largely controlled by vibrational properties rather than specific mineral chemistry. Similar conclusions have been reached from astrochemical and mineral physics studies, which demonstrate that zero-point energy differences largely dictate isotopic partitioning across a wide range of materials. This near-universality suggests that gas–grain isotopic exchange may have operated as a pervasive mechanism in the early solar nebula.^11,14^

**Figure 3:**
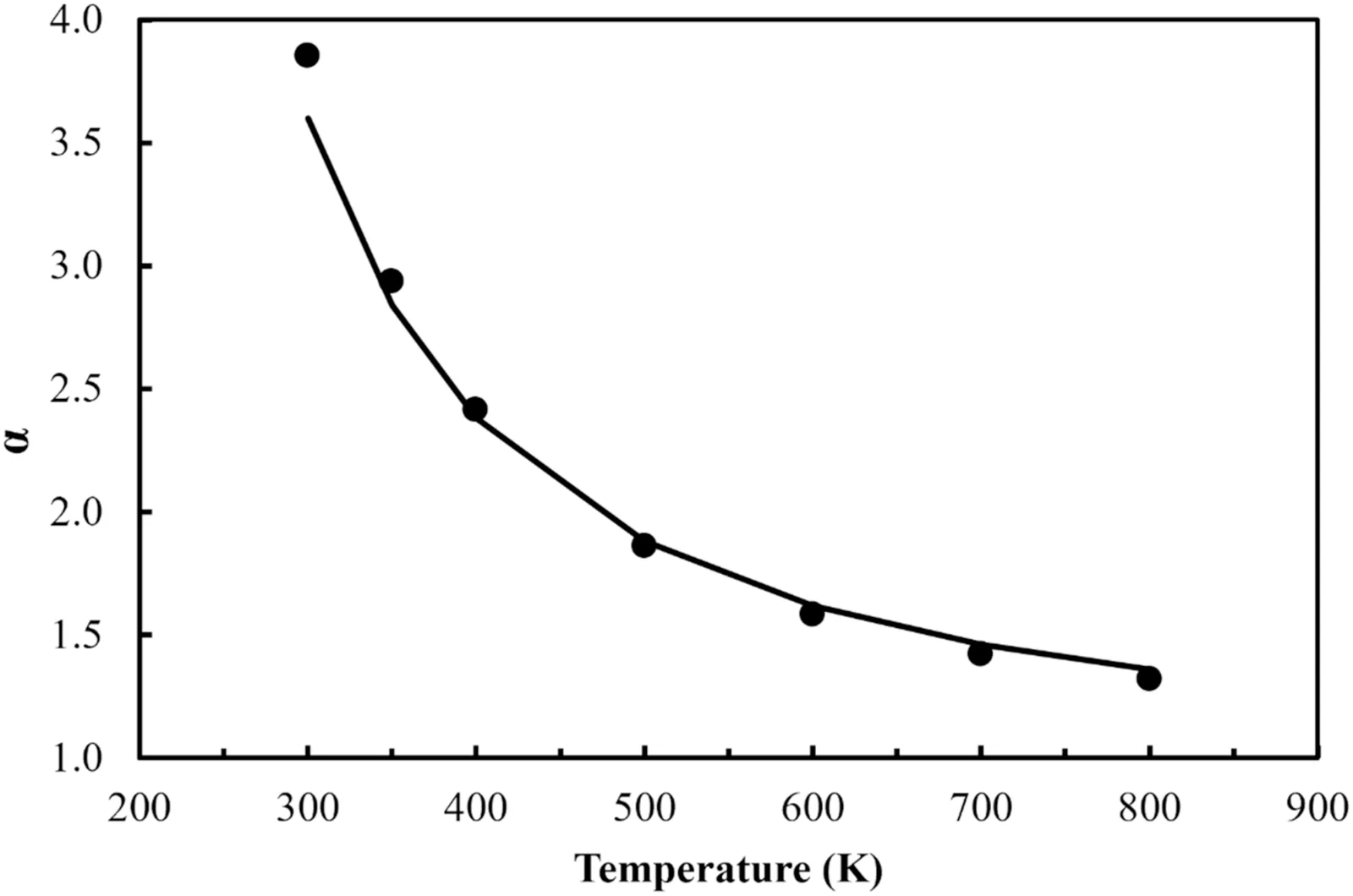
Calculated deuterium enrichment factor, α, (solid spheres = calculated value, line = fitted value) of chemisorbed hydroxyls on olivine surface as a function of temperature.

**Figure 4:**
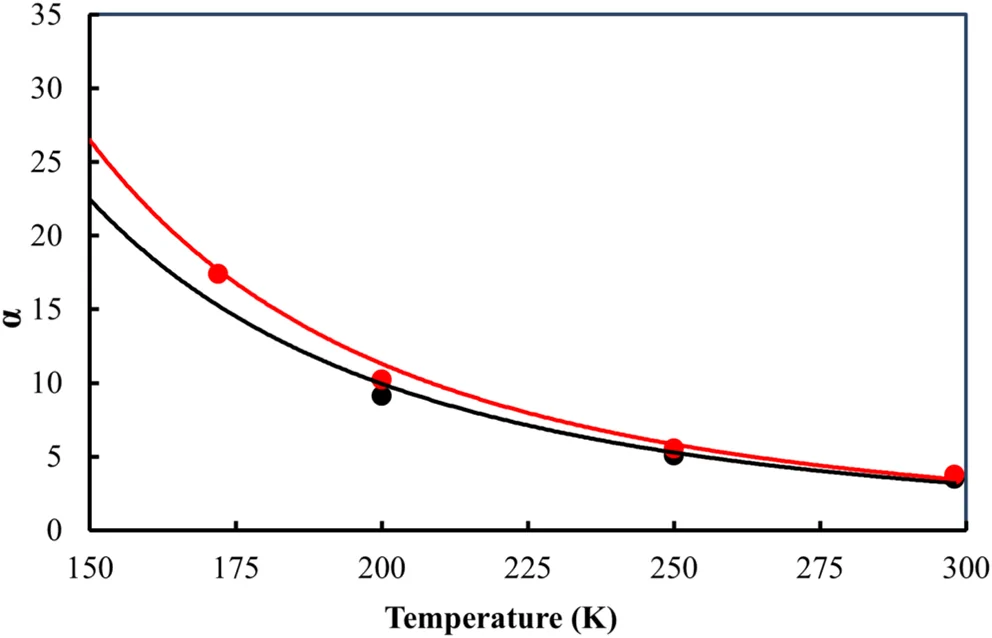
Calculated deuterium enrichment factor, α, in benzylic (red line) and aromatic hydrocarbons (black line). The observed α values in the hydrocarbons extracted from the insoluble organic matter (IOM) of primitive carbonaceous chondrites^31^ are shown by filled symbols.

**Figure 5:**
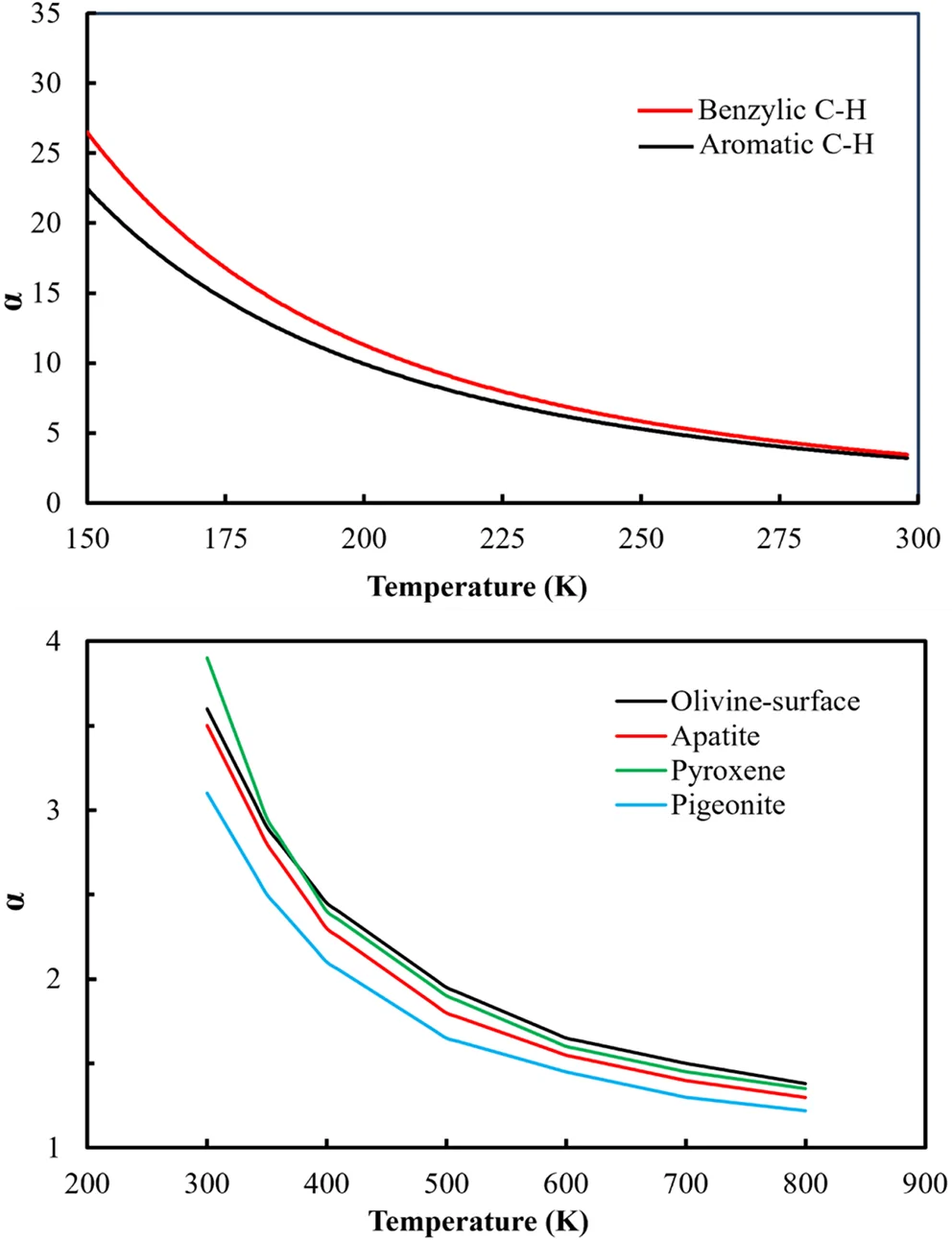
Comparison of the calculated deuterium enrichment factor, α, in several minerals and organics as a function of temperature.

A key result is that enrichment factors comparable to those observed for terrestrial water and inner solar system materials are achieved at temperatures of ∼175–300 K. Thus, equilibrium thermodynamics alone demonstrates that deuterium enrichment of nebular hydrogen can arise naturally during interaction with condensed phases at sufficiently low temperatures. These temperatures are also consistent with constraints derived from isotopic studies of meteoritic organics and protoplanetary disk models.^8^

### 3.2 Comparison with Observed Isotopic Compositions

The calculated fractionation curves (Figure 4) provide a direct basis for comparison with measured D/H ratios in extraterrestrial and terrestrial materials.^31^ The enrichment factors inferred for hydrocarbons in carbonaceous chondrites are in good agreement with the theoretical curves for aromatic and benzylic structures when equilibration temperatures of ∼175–300 K are assumed. This agreement indicates that the observed isotopic compositions of meteoritic organics may have been established through low-temperature gas–grain exchange in the solar nebula. Independent analyses of meteoritic organic compounds show significant deuterium enrichment consistent with formation in cold environments involving grain-surface chemistry.^13^ The measured bulk D/H ratio of meteoritic IOM should be interpreted as a weighted average of multiple site-specific fractionation factors. The benzene and ethylbenzene calculations therefore serve as representative bounds rather than complete structural analogues of meteoritic organic matter.

Similarly, the calculated enrichment factor for chemisorbed hydroxyl groups on olivine reaches values of ∼3.5–3.6 at ∼300 K, comparable to the enrichment of terrestrial mantle water relative to the solar nebula. This result is significant because it directly addresses a major criticism of adsorption-based models for the origin of Earth’s water, namely that such mechanisms would yield nebular (low) D/H ratios. The present results demonstrate that equilibrium isotopic fractionation can elevate the D/H ratio of adsorbed water to terrestrial values suggests that indigenous nebular processes may have contributed substantially alongside external sources such as carbonaceous chondrites. These findings are consistent with emerging isotopic constraints suggesting that significant hydrogen incorporation occurred during accretion rather than through later delivery.^7^

### 3.3 Implications for Adsorption-Mediated Water Accretion

The above thermodynamic results can be directly applied to the model of chemisorption-mediated water accretion on olivine grains developed in Asaduzzaman et al.^8^ In that study, dissociative adsorption of water on olivine surfaces (Figure 2 and S1) was shown to supply several ocean equivalents of water to the growing Earth. However, that model did not explicitly address the isotopic composition of the adsorbed water.

The present calculations demonstrate that such chemisorbed hydroxyls would have undergone isotopic exchange with the surrounding nebular hydrogen, leading to temperature-dependent deuterium enrichment. At temperatures near 300 K, the calculated enrichment factor is consistent with terrestrial values, implying that adsorption and isotopic equilibration could have occurred concurrently during the late stages of accretion. Recent quantum mechanical studies of adsorption on silicate grain surfaces further support the efficiency of surface-mediated reactions and isotopic partitioning in nebular environments. Therefore, adsorption-driven accretion provides a plausible mechanism for explaining both the abundance and isotopic composition of the Earth’s water within a unified framework.

The present calculations employ ideal hydroxylated olivine surfaces. Real nebular grains likely contain defects, amorphous rims, irradiation products, and mixed mineral assemblages. These factors may alter local vibrational frequencies and exchange kinetics. Consequently, the reported fractionation factors should be viewed as representative values for idealized systems.

### 3.4 Kinetic Feasibility of Isotopic Equilibration

The attainment of equilibrium isotopic fractionation depends not only on thermodynamics but also on the rate of exchange processes. Although direct kinetic data for reaction (a) are lacking, insight into the feasibility of isotopic equilibration can be obtained from experimental measurements of D–H interdiffusion in olivine at 750–900 °C, 2 GPa that were collected by exchanging D from a fluid source (nominal mixture of 90% D_2_O and 10% H_2_O) with H in olivine.^32^

Using the experimentally reported interdiffusion coefficient (*D*(D–H) = 10^–4.9^exp­(−16839/*T*) m^2^/s) and the Einstein relation χ ∼ √(2*D*t) for diffusion length, the effective depth of hydrogen isotope exchange within olivine has been estimated as a function of temperature over a time scale of 5 Myr, which corresponds to the lifetime of the solar nebula. The results (Figure 6) indicate that exchange distances of ∼5–400 Å can be achieved at temperatures of ∼260–300 K. These distances are sufficient to ensure equilibration within the chemisorbed surface layer (∼5 Å thick) and part of the near-surface crystal structure. Experimental and modeling studies further demonstrate that hydrogen diffusion and isotope exchange in olivine are efficient even at relatively low temperatures, supporting the plausibility of equilibration under nebular conditions.^15^

**Figure 6:**
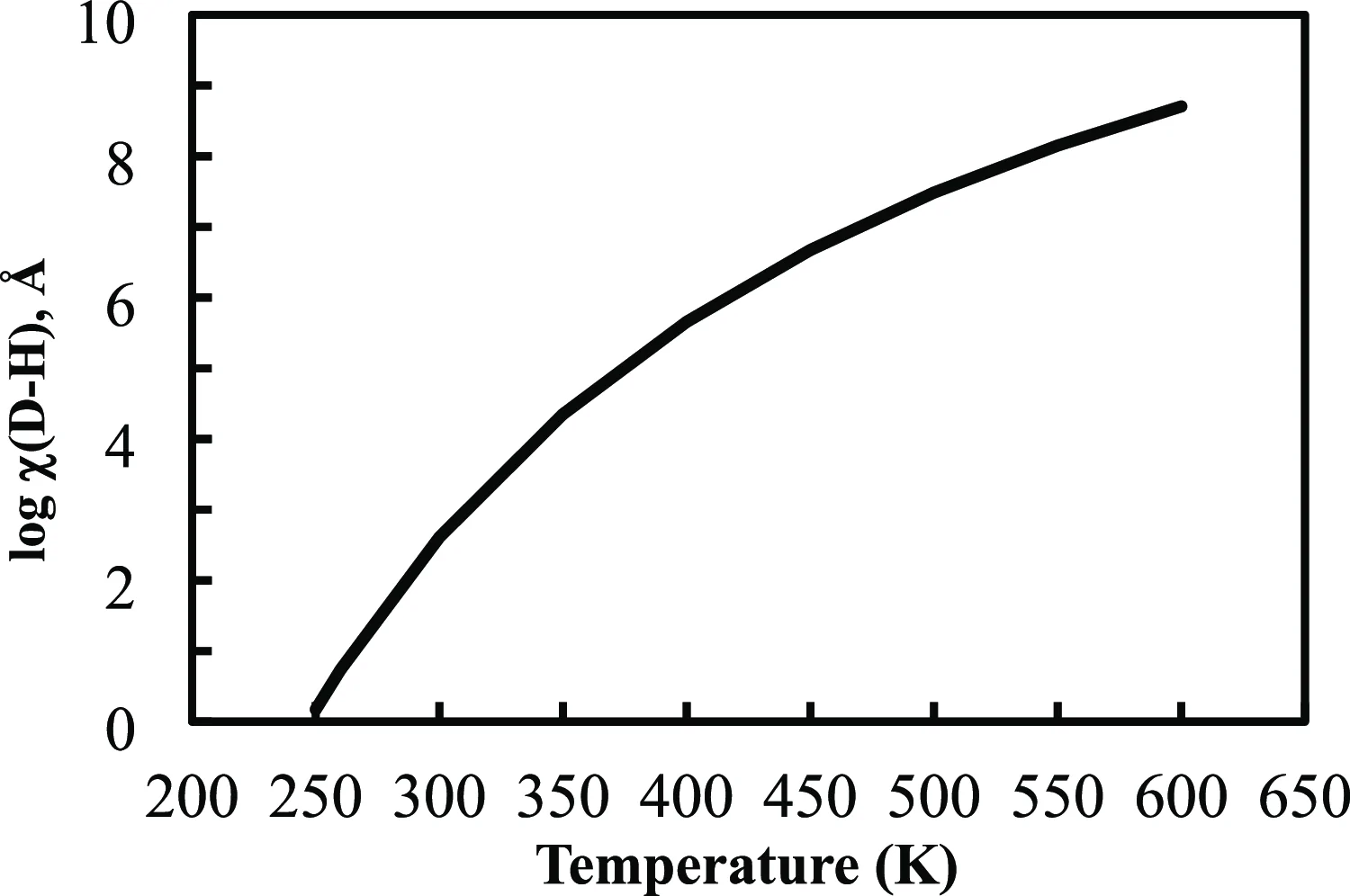
Logarithm of the effective depth of diffusive D–H exchange, χ, within olivine with a nebular gas in 5 Myr.

It is noteworthy that these estimates are conservative because diffusion coefficients increase with decreasing pressure, and the nebular environment is characterized by significantly lower pressures than those of laboratory experiments. In addition, the less compact structure of the chemisorbed layer relative to crystalline olivine likely facilitates even faster isotopic exchange. These calculations suggest that equilibrium D–H fractionation may be kinetically plausible under nebular conditions. However, the estimates involve extrapolation of experimentally determined Arrhenius relations over several hundred degrees, and the resulting exchange depths should therefore be regarded as order-of-magnitude approximations rather than quantitative predictions. Surface isotope exchange may also be governed by O–H/O–D bond breaking and reforming processes that are distinct from bulk diffusion. Presence of surface defects may also impact on the exchange rate. Direct measurements under nebular conditions are currently unavailable and constitute an important target for future laboratory studies.

### 3.5 Implications for Inner Solar System Bodies

Experimental studies of the modal abundance of minerals as a function of P and T using the Martian bulk composition showed that the Martian mantle is similar to the Earth’s upper mantle and has ∼60 wt % olivine or its high pressure polymorphs.^33^ Thus, accretion of water primarily via chemisorption on olivine surfaces would lead to similar water concentration and D/H ratio in the Martian mantle as in the Earth. A recent study of hydrogen isotopic ratio of a variety of phases including olivine from the meteorite Tissint, which is the latest Martian meteorite fall, yields δD of −218 (±96)‰^34^ that is comparable to that determined for melt inclusions in olivine derived from terrestrial mantle.^35^ This similarity in the δD values of the terrestrial and Martian mantle samples is compatible with my model of water accretion in the inner solar system objects via chemisorption, which should be a general nebula process.

The D/H ratios of apatite and low-Ca clinopyroxene extracted from the basaltic eucrite in the asteroid Vesta^36^ were inherited from the basaltic magma since there is practically no hydrogen isotopic fractionation at the crystallization temperatures of minerals. The basaltic magma had, in turn, inherited the D/H ratio of the precursor grains, primarily olivine, without any measurable fractionation. If the D/H ratio of the precursor grains were determined by isotopic fractionation with the solar nebula, then they should have similar enrichment factor, α, as the Earth. One would thus expect, and as observed, the D/H ratio of the apatite in eucrites to be comparable to the terrestrial value.

The α vs *T* relation illustrated in Figure 5 shows that apatite, orthopyroxene, pigeonite, organics (benzylic and aromatic hydrocarbons) and chemisorbed hydroxyls in olivine are quite similar. This observation implies that the O–H vibrational frequencies in solids that affect gas-grain hydrogen isotopic fractionation are similar in different types of chemical environments in the solid states. Thus, in general, a similar α vs T relation for gas-grain hydrogen isotopic fractionation are expected. Consequently, if the gas-grain equilibration temperatures are comparable in different inner solar system objects, then similar enrichment factors should be expected for all such bodies, as seems to be the case. Thus, in my model, the similarity between the isotopic enrichment factor of CCs and the Earth is not primarily due to delivery of water by the CCs but is a natural consequence of analogous gas-grain hydrogen isotopic fractionation of organics and inorganics. The observed D/H ratios imply a low temperature, ∼175–300 K, for hydrogen isotopic equilibration between the solar nebula and dust grains that formed CCs and the Earth, as well as the asteroid Vesta. However, significantly lower enrichment factors may be found in the deep-seated samples in the Earth’s interior as a result of higher accretion and isolation temperature.

Carbonaceous chondrites are known to have clay minerals, and the D/H ratio of the latter could have a significant effect on the overall hydrogen isotopic ratio of these meteorites. The clay minerals had formed by the alteration effect of water. Thus, if the water was derived directly from the solar nebula, then the D/H ratio of the clay minerals would be the same as that determined by isotopic fractionation between water and hydrogen in the nebular environment. The results illustrated in Figure 5 show that the organics and the water in clay minerals in the CCs should have similar enrichment factors in equilibrium with the solar nebula at *T* > 350 K.

The similarity of the calculated α - T relations across a wide range of materials has important implications for the origin of water in the Earth, Mars, asteroid Vesta, and carbonaceous chondrites. Because the fractionation behavior is largely insensitive to detailed chemical composition, anybody that experienced gas–grain equilibration at similar temperatures would develop comparable D/H ratios.

For the Earth, the results suggest that the observed enrichment reflects equilibration at temperatures near ∼300 K during the later stages of accretion. The strong temperature dependence of α further implies that the D/H ratio of terrestrial reservoirs should be heterogeneous, with deeper regions accreted at higher temperatures exhibiting lower enrichment.

For Mars, the similarity in mineralogical composition, particularly the abundance of olivine, implies comparable water incorporation and isotopic evolution. Observations of Martian meteorites showing mantle-like D/H ratios are consistent with this prediction.

For Vesta and carbonaceous chondrites, the D/H ratios measured in apatite, pyroxene, and organic matter can likewise be explained by equilibration with the solar nebula at low temperature.

The convergence of enrichment factors across these different bodies suggests that the similarity in their isotopic compositions does not require large-scale delivery of water from carbonaceous chondrites but instead reflects a common nebular fractionation process.

### 3.6 Effect of Desorption during Accretion

Partial desorption of water could have taken place during the course of accretion of the Earth. The effect of desorption on the D/H ratio of the residual water on the surfaces of the olivine grains may be calculated using the Polanyi–Wigner relation that has been discussed in a number of studies (e.g., Reider et al.)^37^ and is as follows

6 $$\frac{{\mathrm{Ra}te}_{OH}}{{Rate}_{OD}}\approx \beta \exp\left(-\frac{\Delta E}{\mathrm{RT}}\right)$$

where β is the ratio of frequency factors of OH and OD and Δ*E* is the difference between the activation energies of desorption of OD and OH (*E*_OD_ – *E*_OH_). The parameter β may be approximated as the ratio of vibrational frequencies, ν, of the adsorbed OH and OD, whereas Δ*E* is essentially the difference between the zero-point energies of these species. Since the force constants of these species are the same under Born–Oppenheimer approximation (McQuarrie),^38^ the parameter β equals *m̅*(OD)*/m̅*(OH), where *m̅* is the reduced mass. From the vibrational frequencies of OH and OD, the calculated Δ*E* is 8.45 kJ/mol. Using these values of β and Δ*E*, the relative desorption rates of OH and OD from the last expression have been calculated. Because *E*_OD_ exceeds *E*_OH_ due to the lower zero-point energy of O–D bonds, OH-bearing species desorb more rapidly than OD-bearing species. The resulting kinetic isotope effect preferentially enriches the residual surface reservoir in deuterium.

Using eq 7, the calculated desorption rate of OH is ∼4–6 times greater than that of OD in the temperature range of 1000–800 K (with the ratio increasing with decreasing temperature). Step heating of three out of four Martian samples at 423–1273 K showed progressive decrease of D/H ratio of the released water with increasing temperature that suggests preferential loss of H relative to D with increasing temperature.^39^ Also, muscovite crystals irradiated at 30 keV in an electron microscope have been found to develop quite significant bulk deuterium enrichment.^40^ The simplest explanation of this observation is the preferential loss of H relative to D during dehydration of muscovite caused by the thermal effect of electron irradiation.^13^

It may be argued that during the accretion process, partial desorption of water had taken place by (a) fracturing of the growing planetesimals resulting from their mutual impacts and (b) also by the heat produced by the decay of short-lived radioactive isotope ^26^Al (*t*_1/2_ ∼ 730,000 y) to ^26^Mg. With respect to (a), the fracturing would have mostly taken place at the regions of contact between the impacting planetesimals. Since these fractures, or at least a major fraction thereof, were not open to the ambient atmosphere, they would not have provided pathways for the escape of water. With respect to (b), desorption of water would have taken place within the interior of asteroids when the temperature exceeded the initial chemisorption temperature of water, and up to the extent prescribed by the Langmuir isotherm corresponding to the interior temperature. However, since the surface temperatures of the planetesimals were buffered by the cold nebular atmosphere, the desorbed water would have gotten trapped by the cold outer segments of the planetesimals unless it encountered fractures connecting to an open surface. Nevertheless, some desorption had very likely taken place during the accretion process of the Earth and thus caused depletion of the water budget that was accreted from the solar nebula. In that case, the D/H ratio of the residual hydroxyls would have increased, depending on the extent of desorption.

### 3.7 Synthesis: Resolution of the D/H Constraint

A key challenge to models of adsorption-mediated water delivery has been the apparent incompatibility between nebular hydrogen and the elevated D/H ratio of terrestrial water. The results of this study provide a plausible mechanism to resolve this issue by demonstrating that equilibrium gas–grain isotopic fractionation provides a natural mechanism for enriching deuterium in condensed phases.

The combined effects of (i) thermodynamic preference of deuterium for solids, (ii) low-temperature equilibration in the solar nebula, and (iii) kinetically feasible exchange processes lead to enrichment factors consistent with those observed for the Earth and other inner solar system objects. Consequently, adsorption of water onto mineral grains from the solar nebula plausibly accounts for both the quantity and isotopic composition of terrestrial water without invoking large-scale delivery from external reservoirs.

Using a representative solar nebula D/H ratio of ∼2 × 10^–5^, the calculated enrichment factors (α ≈ 3.5–8 over 175–300 K) correspond to absolute D/H values spanning ∼7 × 10^–5^ to ∼1.6 × 10^–4^. These values overlap with those of the Earth’s mantle, SMOW, carbonaceous chondrites, Martian mantle samples, and eucrites, indicating that equilibrium gas–grain fractionation can quantitatively reproduce the observed isotopic compositions.

## 4 Conclusion

Building on earlier work demonstrating chemisorption-mediated incorporation of water into olivine (Asaduzzaman et al.),^8^ the present study establishes a viable alternative for explaining both the abundance and isotopic composition of water in the Earth and other inner solar system bodies. The central result is that equilibrium gas–grain hydrogen isotopic fractionation in the solar nebula produces a temperature-dependent enrichment of deuterium in condensed phases investigated here (olivine hydroxyls, apatite, orthopyroxene, pigeonite, and representative organic compounds), with enrichment increasing systematically as temperature decreases.

The calculations show that chemisorbed hydroxyls on olivine surfaces, structural hydroxyls in silicate minerals, and hydrogen in organic compounds all exhibit broadly similar fractionation behavior. At temperatures of ∼175–300 K, the predicted enrichment factors match those observed for terrestrial water, carbonaceous chondrites, Mars, and asteroid Vesta. This convergence indicates that the isotopic similarity among these bodies can arise naturally from gas–grain equilibration under comparable nebular conditions, without necessarily requiring that all water be supplied by large-scale delivery from carbonaceous chondrites.

A key implication of this work is the resolution of a major criticism of adsorption-based models. Although nebular hydrogen has a near-solar D/H ratio, the results demonstrate that isotopic exchange between gas and grain surfaces can substantially elevate the D/H ratio of adsorbed water. When coupled with kinetic constraints indicating that equilibration may have been approached within the lifetime of the solar nebula, chemisorption emerges as a viable and self-consistent mechanism for producing both the quantity and isotopic signature of terrestrial water. The results demonstrate that equilibrium gas-grain fractionation is a viable mechanism capable of producing terrestrial-like D/H enrichments if isotopic exchange proceeded sufficiently rapidly.

The strong temperature dependence of the enrichment factor further implies that planetary interiors should exhibit spatial variability in D/H ratios, reflecting differences in equilibration and isolation temperatures during accretion. This prediction is consistent with emerging evidence for isotopic heterogeneity in the Earth’s mantle and provides a testable constraint for future geochemical studies.

More broadly, these results highlight gas–grain interactions in the solar nebula as a viable alternative process governing water incorporation and isotopic evolution across the inner solar system. The observed D/H ratios of the Earth, Mars, Vesta, and carbonaceous chondrites can thus be interpreted as the outcome of a common thermodynamic mechanism operating over similar temperature ranges, rather than as evidence for multiple distinct sources of water.

## Supplementary Material



## Notes

Vibrational frequency of condensed phases and Cartesian coordinates of representative clusters and organics (PDF)
