# Supplementary material for: Hydrogen Isotopic Fractionation in the Solar Nebula and Implications for Water in Earth and Inner Solar System Bodies
Source: ACS Earth Space Chem. 2026 Sep 24;10(10):2326–34. doi: 10.1021/acsearthspacechem.6c00183 (PMC13679083; doi:10.1021/acsearthspacechem.6c00183)
Supplement: Supplementary file 1 [file sp6c00183_si_001.pdf]

**Hydrogen isotopic fractionation in the solar nebula and implications for water in Earth and inner solar system bodies**


Abu Asaduzzaman*

School of Science, Engineering and Technology, Penn State Harrisburg, Middletown, PA 17057, USA

*aua1309@psu.edu


**Supporting Information**

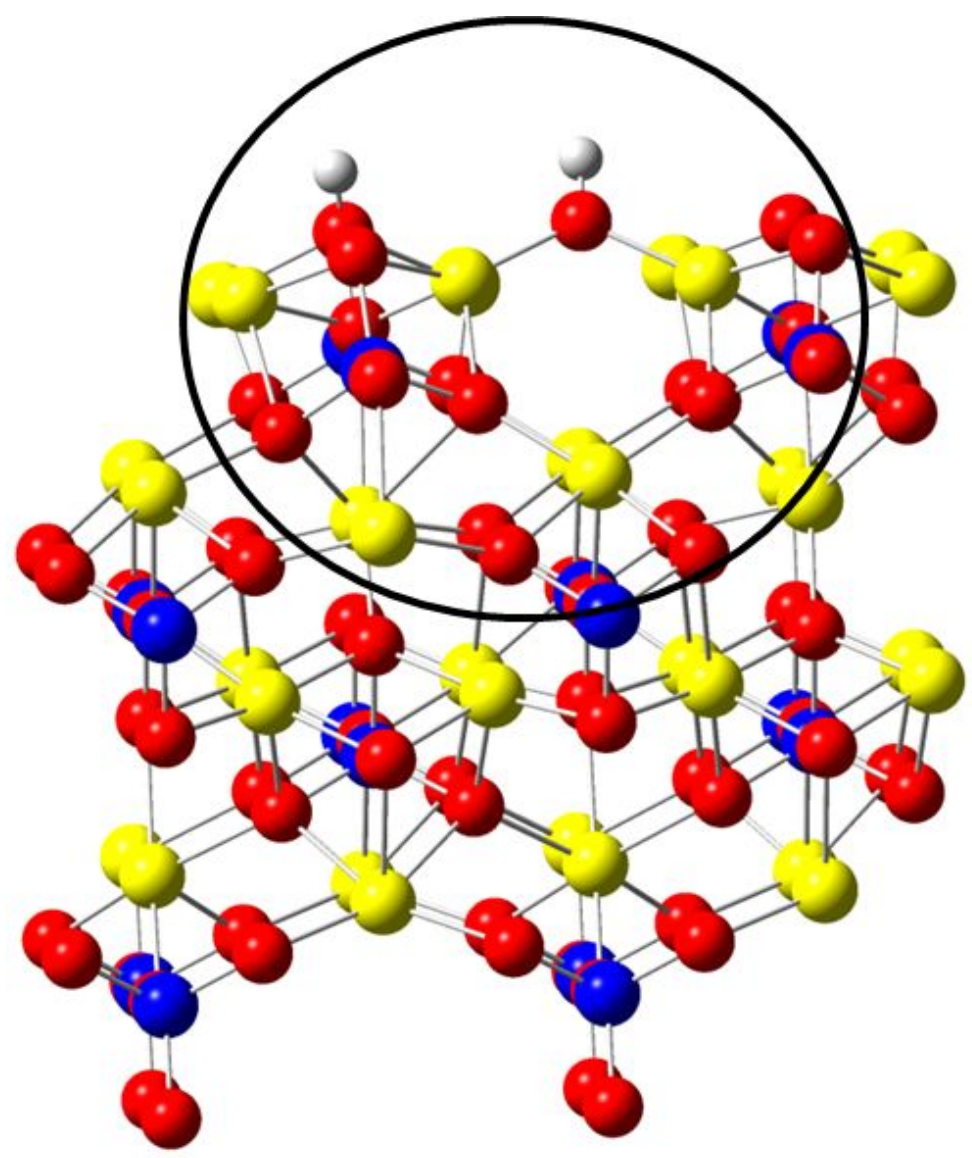

**Figure S1**: Ball and stick illustration of a VASP-optimized olivine surface. The circle denotes the cluster that was carved out for calculations using Gaussian. The ball colors for the atoms are as follows; yellow: Mg, red: O, blue: Si, white: H.

**Table S1.** Calculated vibrational frequency ($cm^{-1}$) for condensed phase and gas species. Corresponding experimental values are included in the parenthesis whenever available.

| Vibrational Frequency ($cm^{-1}$) | |
|---|---|
| OH | OD |
| **Chemisorbed surface** | |
| 275.8765 | 243.0462 |
| 290.1185 | 273.9535 |
| 446.6573 | 358.7952 |

| | |
|---|---|
| 475.3812 | 462.1374 |
| 840.3287 | 628.8666 |
| 3704.296 | 2696.462 |
| **Apatite** | |
| 231.4 | 225 |
| 319.8 | 309.5 |
| 322.7 | 313 |
| 817.7 | 597.8 |
| 825.6 | 601.9 |
| 3566.7 (3572)[1] | 2594.2 |
| **Pyroxene** | |
| 144.984 | 140.5166 |
| 288.8 | 278.4 |
| 712.1192 | 521.6 |
| 728.08 | 664.07 |
| 1073.6 | 836.06 |
| 3222.2 | 2345.8 |
| **Pigeonite** | |
| 211.8635 | 207.6282 |
| 456.9643 | 429.9112 |
| 567.7987 | 523.6105 |
| 729.7223 | 560.2399 |
| 1053.525 | 784.78 |
| 3567.794 | 2597.141 |
| **Gas molecules** | |
| $H_2$ = 4421 (4401)[2]<br>HD = 3829 (3813)[2]<br>$D_2$ = 3127 (3116)[2] | |

In addition, the ratio of the calculated vibrational frequencies of chemisorbed OD and OH is found to be 0.727, which is the same as $(\overline{m}(OH)/\overline{m}(OD))^{1/2}$, where $\overline{m}$ is the reduced mass. This is what is expected from theory since ν is inversely proportional to the reduced mass, with the proportionality constant being given by $(\sqrt{k_f})/2\pi$, where $k_f$ is the force constant and is the same for OH and OD under Born-Oppenheimer approximation.[3]

**Table S2.** Cartesian coordinates of representative clusters and ethylbenzene.

**Hydroxylated olivine surface**

| | | | |
|---|---|---|---|
| Mg | 0.08130945047831 | 0.11817557112949 | 10.90558151302904 |
| Mg | 6.08430530997522 | 0.11813184578819 | 10.90526785015115 |
| Mg | 0.15956137039746 | 4.98141774829676 | 10.86040375388539 |
| Mg | 6.16245146446911 | 4.98121759024831 | 10.86021699858811 |
| Mg | 2.92019469002478 | 0.11813184578819 | 10.90526785015115 |
| Mg | 8.92319054952169 | 0.11817557112949 | 10.90558151302904 |
| Mg | 2.84204853553089 | 4.98121759024831 | 10.86021699858811 |
| Mg | 8.84493862960255 | 4.98141774829676 | 10.86040375388539 |
| O | 7.50365755139048 | 4.25712544801836 | 12.14200211480986 |
| O | 1.50084244860952 | 4.25712544801836 | 12.14200211480986 |
| O | 7.50358352556070 | -0.03148789372973 | 12.23425190572354 |
| O | 1.50091647443930 | -0.03148789372973 | 12.23425190572354 |
| O | 3.17492935259083 | 3.46043353323162 | 9.42183870065432 |
| O | 9.17750242725274 | 3.46048086586035 | 9.42257077962768 |
| O | 3.19547991068548 | 8.30307549403007 | 9.36592455125683 |
| O | 9.19842390668390 | 8.30327903683553 | 9.36591620862887 |
| O | 5.82957064740917 | 3.46043353323162 | 9.42183870065432 |
| O | 11.83299757274727 | 3.46048086586035 | 9.42257077962768 |
| O | 5.80902008931452 | 8.30307549403007 | 9.36592455125683 |
| O | 11.81207609331610 | 8.30327903683553 | 9.36591620862887 |
| O | 4.50225000000000 | 3.73064761117665 | 11.63148865900721 |
| O | 10.50525000000000 | 3.73172902033006 | 11.63209508441666 |
| O | 4.50225000000000 | 8.32205313427469 | 11.59498978766085 |
| O | 10.50525000000000 | 8.32168227665218 | 11.59563924940490 |
| O | 4.50225000000000 | 1.25606516797316 | 10.49352926204950 |
| O | 10.50525000000000 | 1.25636895182283 | 10.49500593840919 |
| O | 4.50225000000000 | 5.95449391068551 | 10.17307188085205 |
| O | 10.50525000000000 | 5.95506868068898 | 10.17283730544155 |
| Si | 4.50225000000000 | 2.83148990376904 | 10.12774636528506 |
| Si | 10.50525000000000 | 2.83196759851230 | 10.12875803447747 |
| Si | 4.50225000000000 | 7.55370151374341 | 10.00067161356492 |
| Si | 10.50525000000000 | 7.55411993606803 | 10.00047251798562 |
| H | 4.50225000000000 | 3.22571197708372 | 12.46390983077003 |
| H | 4.50225000000000 | 7.76153699633857 | 12.39337622849705 |
| H | 1.50084351880880 | 4.47253261691169 | 13.09214722917923 |
| H | 7.50365648119120 | 4.47253320826767 | 13.09214722917923 |
| H | 1.50098928395921 | 0.40041111615295 | 13.10871630699828 |
| H | 7.50351071604080 | 0.40041111615295 | 13.10871630699828 |
| H | 10.50525000000000 | 3.22637131631080 | 12.46421018106285 |
| H | 10.50525000000000 | 7.76122196955127 | 12.39391563614487 |

**Apatite**

| | | | |
|---|---|---|---|
| Ca | 5.89212300 | 10.17623400 | 1.71596700 |
| Ca | 2.34751100 | 8.15897400 | 1.71596700 |
| Ca | 3.53748300 | 10.19084500 | 5.13561700 |

| | | | |
|---|---|---|---|
| Ca | 5.86681600 | 6.09787900 | 1.71596700 |
| Ca | 7.05678900 | 8.12975100 | 5.13561700 |
| Ca | 3.51217700 | 6.11249100 | 5.13561700 |
| P | 1.09491000 | 8.39137300 | 5.14344100 |
| P | 6.71968700 | 11.14481900 | 5.14344100 |
| P | 3.11244700 | 11.39182900 | 1.72379100 |
| P | 6.29185300 | 4.89689500 | 5.14344100 |
| P | 2.68461200 | 5.14390600 | 1.72379100 |
| P | 8.30939000 | 7.89735200 | 1.72379100 |
| H | 4.70215000 | 8.14436200 | 2.95084000 |
| H | 4.70215000 | 8.14436200 | 6.35475600 |
| O | 6.75367000 | 10.25816600 | 6.39281000 |
| O | 6.67982700 | 10.22128900 | 3.91927500 |
| O | 1.91463600 | 8.81862500 | 3.91927500 |
| O | 1.84578200 | 8.86412900 | 6.39281000 |
| O | 3.89730200 | 10.97793300 | 2.97316000 |
| O | 3.89232700 | 10.89554400 | 0.49962500 |
| O | 5.50699800 | 5.31079200 | 6.39281000 |
| O | 5.51197300 | 5.39318000 | 3.91927500 |
| O | 7.48966400 | 7.47009900 | 0.49962500 |
| O | 7.55851700 | 7.42459600 | 2.97316000 |
| O | 2.65063000 | 6.03055900 | 2.97316000 |
| O | 2.72447300 | 6.06743600 | 0.49962500 |
| O | 4.70215000 | 8.14436200 | 1.96924000 |
| O | 4.70215000 | 8.14436200 | 5.38889000 |

**Pyroxene**

| | | | |
|---|---|---|---|
| Al | 6.71653043 | 4.30261149 | 7.03155148 |
| Mg | 6.84788855 | 5.78383330 | 4.45854066 |
| Mg | 6.82169548 | 4.23583106 | 1.89673217 |
| Mg | 2.25865442 | 3.00536762 | 1.87611960 |
| Mg | 2.24467827 | 4.48290582 | 4.39759631 |
| Mg | 6.79814268 | 0.18993109 | 4.44214100 |
| Mg | 2.23014416 | 1.38228607 | 4.45999152 |
| Mg | 6.87131142 | 0.13269668 | 9.62787031 |
| Mg | 2.32953318 | 3.01620496 | 7.00800004 |
| O | 3.33716658 | 2.97365339 | 0.16147052 |
| O | 5.65839341 | 4.41814203 | 0.22919944 |
| O | 5.53335089 | 1.86551495 | 4.45448028 |
| O | 7.87158112 | 4.27050968 | 3.59329544 |
| O | 8.12649047 | 1.72115866 | 3.13582803 |
| O | 5.76892578 | 5.77881121 | 2.75606560 |
| O | 3.42739923 | 4.35696094 | 2.74140340 |
| O | 3.34287072 | 1.42837843 | 2.77075786 |
| O | 5.53743561 | 2.48817317 | 1.80022313 |
| O | 3.59140044 | 6.28125901 | 4.31516717 |

| | | | |
|---|---|---|---|
| O | 3.29327886 | 3.00755691 | 5.27124655 |
| O | 5.73369422 | 4.59455013 | 5.56509465 |
| O | 5.56413397 | 1.93000175 | 9.54525922 |
| O | 7.72838326 | 4.12068162 | 8.58057688 |
| O | 8.16597123 | 1.59210327 | 8.34514100 |
| O | 5.79529456 | 5.69760397 | 7.94595282 |
| O | 3.46249058 | 4.31469886 | 7.99280132 |
| O | 3.61156550 | 6.81936756 | 6.90697356 |
| O | 7.90830688 | 5.64311119 | 6.29852727 |
| O | 3.40283176 | 1.44550786 | 7.89429129 |
| O | 5.68055588 | -0.01557915 | 7.98022016 |
| O | 5.78256678 | 2.53477505 | 7.01529230 |
| O | 7.89388900 | 0.12112945 | 6.13028468 |
| O | 8.14191284 | 2.70373589 | 5.76754554 |
| O | 5.78183290 | 7.37978572 | 5.35178350 |
| O | 3.40616232 | 0.03422074 | 5.37318486 |
| O | 3.59690068 | 6.30565905 | 9.48788433 |
| Si | 4.94752575 | 2.98760477 | 0.31732259 |
| Si | 8.59825984 | 2.96986792 | 4.16382207 |
| Si | 4.15164385 | 5.76819636 | 2.82361781 |
| Si | 4.96673699 | 1.42278199 | 2.97760049 |
| Si | 4.15399794 | 5.74183020 | 8.02479106 |
| Si | 5.01197217 | 1.43571153 | 8.04870073 |
| Si | 8.62327497 | 1.40457310 | 6.73178324 |
| Si | 4.16443358 | 7.37205229 | 5.42112867 |
| H | 5.69863019 | 2.29276958 | 6.00698708 |
| H | 4.90820210 | 4.09167041 | 5.39700718 |
| H | 4.15447392 | 2.56998357 | 4.99558748 |

**Ethylbenzene**

| | | | |
|---|---|---|---|
| C | -0.22621059 | -2.31893677 | 0.00000000 |
| C | -0.09104691 | -1.63139648 | 1.21364817 |
| C | 0.17923635 | -0.25630778 | 1.21364786 |
| C | 0.31437562 | 0.43123675 | 0.00000000 |
| C | 0.17923635 | -0.25630778 | -1.21364786 |
| C | -0.09104691 | -1.63139648 | -1.21364817 |
| H | -0.43260793 | -3.36884159 | 0.00000000 |
| H | -0.19423929 | -2.15634991 | 2.14029548 |
| H | 0.28242157 | 0.26864749 | 2.14029492 |
| H | 0.28242157 | 0.26864749 | -2.14029492 |
| H | -0.19423929 | -2.15634991 | -2.14029548 |
| C | 0.61138739 | 1.94232378 | 0.00000000 |
| C | -0.71427520 | 2.72604483 | 0.00000000 |
| H | 1.17510790 | 2.19501256 | -0.87365251 |
| H | 1.17510790 | 2.19501256 | 0.87365251 |

| | | | |
|---|---|---|---|
| H | -0.50790988 | 3.77595595 | 0.00000000 |
| H | -1.27799726 | 2.47335596 | -0.87365034 |
| H | -1.27799726 | 2.47335596 | 0.87365034 |

**References**


1. Engel, G.; Klee, W. E. Infrared Spectra of the Hydroxyl Ions in Various Apatites. *Journal of Solid State Chemistry* **1972**, *5* (1), 28–34.

2. Huber, K. P.; Herzberg, G. Constants of Diatomic Molecules. In *Molecular Spectra and Molecular Structure*; Springer US: Boston, MA, 1979; pp 8–689.

3. McQuarrie, D. A. *Statistical Thermodynamics*; University Science Books, Reprint, 1985.
